# Effects of Short-Term Exposure to Particulate Air Pollutants on the Inflammatory Response and Respiratory Symptoms: A Panel Study in Schoolchildren from Rural Areas of Japan

**DOI:** 10.3390/ijerph13100983

**Published:** 2016-09-30

**Authors:** Masanari Watanabe, Hisashi Noma, Jun Kurai, Hiroyuki Sano, Degejirihu Hantan, Masaru Ueki, Hiroya Kitano, Eiji Shimizu

**Affiliations:** 1 Department of Respiratory Medicine and Rheumatology, Faculty of Medicine, Tottori University, 36-1 Nishi-cho, Yonago 683-8504, Japan; junkurajun@gmail.com (J.K.); degujirefu@med.tottori-u.ac.jp (D.H.); eiji@med.tottori-u.ac.jp (E.S.); 2 Department of Data Science, Institute of Statistical Mathematics, 10-3 Midori-cho, Tachikawa, Tokyo 190-8562, Japan; noma@ism.ac.jp; 3 Department of Respiratory Medicine and Allergology, Faculty of Medicine, Kinki University, Ohnohigashi 377-2, Osakasayama 589-0014, Japan; hsano@med.kindai.ac.jp; 4 Center for Promoting Next-Generation Highly Advanced Medicine, Tottori University Hospital, 36-1 Nishi-cho, Yonago 683-8504, Japan; masaruueki@gmail.com; 5 The Board of Directors, Tottori University, 36-1 Nishi-cho, Yonago 683-8504, Japan; hkitano@med.tottori-u.ac.jp

**Keywords:** particulate air pollutants, pro-inflammatory cytokine, respiratory symptom, schoolchildren

## Abstract

The relationship between particulate air pollutants and respiratory symptoms in children has not been consistent among studies, potentially owing to differences in the inflammatory response to different particulate air pollutants. This study aimed to investigate the effect of particulate air pollutants on respiratory symptoms and the inflammatory response in schoolchildren. Three hundred-and-sixty children were included in the study. The children recorded daily respiratory symptom scores for October 2015. In addition, the daily amount of interleukin (IL)-6, IL-8, and tumor necrosis factor (TNF)-α production was assessed in THP1 cells stimulated with suspended particulate matter (SPM), which was collected every day during the study period. Generalized estimating equation logistic regression analyses were used to estimate the associations among respiratory symptoms and the daily levels of SPM, IL-6, IL-8, and TNF-α. Daily SPM levels were not associated with respiratory symptoms or the daily IL-6, IL-8, and TNF-α levels. Conversely, there was a significant association between respiratory symptoms and the daily IL-6, IL-8, and TNF-α levels. These results suggested that the effects of particulate air pollutants on respiratory symptoms in schoolchildren might depend more on the pro-inflammatory response to them than on their mass concentration.

## 1. Introduction

The adverse effects of particulate air pollutants on the respiratory system are highly prevalent and significantly contribute to the morbidity and mortality of lung cancer, pneumonia, asthma, pulmonary embolism, and chronic obstructive pulmonary disease, as well as other organ systems—in particular the cardiovascular system—which are categorized as downstream effects [1,2,3,4]. Exposure to particulate air pollutants is strongly associated with these injurious respiratory health effects, even when adjusted for major risk factors such as cigarette smoking [5]. In children particularly, asthma is one of the most common diseases investigated for the effects of particulate air pollutants on pulmonary function and respiratory symptoms, as well as on hospital admissions and emergency visits [6,7,8]. Conversely, few studies have investigated the effects of particulate air pollutants on pulmonary function and respiratory symptoms in children without asthma [8].

Not all studies have found a relationship between particulate air pollutants and pulmonary function. These differences may be caused by differences in participant characteristics, methods of statistical analysis, and amounts of individual exposure. To estimate the relationship between particulate air pollutants and pulmonary function, almost all studies have focused on the mass concentration of particulate air pollutants categorized according to particle size, such as PM_10_ (particulate matter smaller than 10 μm aerodynamic diameters) or PM_2.5_ (PM smaller than 2.5 μm aerodynamic diameters), which represent median aerodynamic diameters of less than 10 μm and 2.5 μm, respectively. Numerous studies have investigated the effects of individual particulate air pollutants, including polycyclic aromatic hydrocarbons and black carbon, on respiratory health [9,10]. Particulate air pollutants are a mixture of solids and liquid droplets suspended in the air, and are formed from various substances [11]. Previous studies demonstrated that the inflammatory potential of particulate air pollutants had heterogeneity in relation to city and season [12,13,14,15], which might be because different components of particulate air pollutants can influence the pro-inflammatory cytokine response differently [16]. Therefore, differences in the results of studies investigating the association between particulate air pollutants and respiratory health might be attributable to disparities in the inflammatory response caused by particulate air pollutants with different compositions. Our previous study found that the effects of particulate air pollutants on pulmonary function in schoolchildren might be more dependent on the pro-inflammatory response than the mass concentration of pollutants [17].

Exposure to airborne PM increases the concentration of interleukin (IL)-8 in bronchoalveolar lavage fluid samples and elevates IL-8 mRNA expression in bronchial biopsy tissues from healthy and asthmatic subjects [18]. Neutrophils migrate to the airways in patients with allergic disease during the acute inflammation phase following exposure to particulate air pollutants [19,20,21]. Additionally, IL-8 is well known as one of the most important neutrophil chemotaxins present in the lower respiratory tract [22]. These findings suggest that exposure to particulate air pollutants induces the augmentation of neutrophilic airway inflammation depending on IL-8 production. To understand the mechanism underlying the injurious effects of particulate air pollutants on human health, previous studies measured the levels of pro-inflammatory cytokine production to assess cell activation after particulate air pollutant exposure in vitro [23]. Tumor necrosis factor (TNF)-α was the cytokine that was most often selected for analysis, followed by IL-8 and IL-6 [23]. 

In the association between airborne PM and health, including respiratory disorders, the quantity of particulate air pollutants is the most important factor. On the other hand, the effects of particulate air pollutants on respiratory systems may be related to the production of pro-inflammatory cytokines, depending on the types and sources of particulate air pollutants. Our previous studies investigated the relationship between particulate air pollutants and pulmonary function in children with and without asthma [17,24,25]. We also found that the effects of particulate air pollutants on pulmonary function in schoolchildren may be more dependent on pro-inflammatory responses than the mass concentration of air pollutants [26]. However, we have yet to investigate the relationship between particulate air pollutants and respiratory symptoms. Additionally, in Japan, the effects of air pollutants on respiratory symptoms in children both with and without asthma have not been sufficiently investigated. This study was conducted to investigate the association between the concentration of particulate air pollutants and respiratory symptoms in schoolchildren. In addition, the relationship between respiratory symptoms and the daily levels of IL-6, IL-8, and TNF-α production in response to particulate air pollutants was evaluated.

## 2. Materials and Methods

### 2.1. Study Design

In this panel study, the respiratory symptoms of schoolchildren were monitored daily in the morning for the entirety of October 2015. The study was performed in Matsue, the capital city of Shimane Prefecture, southwest Japan. This city has a population of approximately 200,000 individuals and covers an area of 530.2 km^2^. A total of 360 students aged 11–13 years from four of 35 elementary schools in Matsue City were enrolled in 2015. The four elementary schools were within 10 km of each other, and all subjects lived within a radius of 1 km of the schools.

The sex, height, and weight of the children, as well as the occurrence of asthma, allergic rhinitis, allergic conjunctivitis, atopic dermatitis, and food allergies, were recorded in October 2015. The subjects were considered to have asthma if they met any of the following criteria in the past 12 months: diagnosis of asthma by a pediatrician, presence of wheezing, use of asthma medication, and a visit to a hospital for asthma. The subjects were considered to have allergic rhinitis, allergic conjunctivitis, atopic dermatitis, and/or a food allergy if they met any of the following criteria in the past 12 months: diagnosis of any of these conditions by a pediatrician, use of medication for any of these conditions, and a visit to a hospital for any of these conditions. The study was approved by the Ethics Committee of the Faculty of Medicine, Tottori University (approval number 2473). The study was also approved by the Matsue City Board of Education and the Parent Teacher Association (PTA) of each elementary school. The children and their parents were informed by teachers and provided written consent.

#### 2.1.1. Recording of Daily Respiratory Symptoms

From 1 October 2015 to 31 October 2015, each child recorded scores for lower respiratory tract symptoms, including a cough and/or sputum. Scores ranged from 0 (no symptoms) to 2 (severe symptoms), as in previous studies [27,28], and were recorded between 8 AM and 9 AM. All children traveled to school on foot and were potentially exposed to air pollutants. 

#### 2.1.2. Measurement of Air Pollutant Levels 

Suspended PM (SPM) is defined under the National Air Quality Standard as any particle with a diameter of <10 μm, with a 100% cut-off [27,29]. In Japan, the Japanese Ministry of the Environment monitors the levels of SPM instead of PM_10_. The concentrations of SPM, PM_2.5_, sulfur dioxide (SO_2_), nitrogen dioxide (NO_2_), and ozone are monitored by the Japanese Ministry of the Environment in Matsue City. Meteorological variables, including daily temperature, humidity, and atmospheric pressure, were obtained from the Japan Meteorological Agency. These data were used to examine the associations between changes in respiratory symptoms and air pollutant levels. Daily average levels of air pollutants, such as SPM, PM_2.5_, SO_2_, NO_2_, and ozone, were calculated from 6 AM of one day to 5 AM of the next day.

#### 2.1.3. Preparation of Airborne Particles

In one of the four schools enrolled in this study, airborne PM was collected from 1 October 2015 to 31 October 2015 on a 20 × 25 cm quartz filter (2500QAT-UP; Tokyo Dylec, Tokyo, Japan) at a flow rate of 1000 L/min using a high-volume air sampler (HV-1000R; Shibata, Tokyo, Japan) for 24 h from 6 AM to 6 AM, as in our previous study [17]. Prior to sampling, the filters were sterilized using dry heat at 240 °C for 30 min in order to remove endotoxins on the filters. To extract PM from the filter, the filters were treated with 4 mL endotoxin-free distilled deionized water (Sterile water endotoxin free; Wako Pure Chemicals, Osaka, Japan) and an ultrasonic apparatus (BRANSONIC2800; Emerson Japan, Atsugi, Japan) for 60 min [30]. The extraction liquids were filtered through 10-μm filters (pluriStrainer 10 μm; pluriSelect, Leipzig, Germany) to remove PM > 10 μm in diameter. Dissolving solution containing PM < 10 μm in diameter were sterilized at 121 °C for 30 min in an autoclave (Tomy SX-300; Tomy, Tokyo, Japan) and stored in a freezer at −70 °C to prevent growth of bacteria and fungi. 

#### 2.1.4. Cell Culture and Measurement of IL-6, IL-8, and TNF-α Production

The THP1 (ATCC^®^ TIB-202™) human monocyte cell line was cultured in Roswell Park Memorial Institute-1640 medium containing 10% (v/v) fetal bovine serum, 0.05 mM 2-mercaptoethanol, 100 U/mL penicillin, 100 μg/mL streptomycin, and 0.5 μg/mL amphotericin B at 37 °C in a humidified cell culture incubator containing 5% CO_2_. IL-6, IL-8, and TNF-α release stimulated by the collected PM solution were calculated according to our previous study [16]. Briefly, THP1 cells (1 × 10^5^ cells/450 μL/tube) in endotoxin-free tubes (pirotube; Seikagaku, Tokyo, Japan) were exposed for 24 h at 37 °C with solvent only (negative control) or 50 μL of each dissolving solution containing PM < 10 μm in diameter from 1 October 2015 to 31 October 2015. After exposure, the viability of the THP1 cells exceeded 95% in all samples, as assessed using a trypan blue-exclusion test. Subsequently, the supernatants were removed and centrifuged at first 250× *g* to remove floating cells, and then at 2500× *g* to remove the remaining particles. The final supernatants were stored at −70 °C. The concentrations of IL-6, IL-8, and TNF-α were measured using an enzyme‒linked immunosorbent assay (ELISA) kit (R&D Systems, Minneapolis, MN, USA), according to the manufacturer’s protocol, and with endotoxin-free 96-well plates (Toxipet plateLP; Seikagaku, Tokyo, Japan). Samples were run in triplicate and read using an automated ELISA reader (Model 680; Bio-Rad, Philadelphia, PA, USA). The concentrations of IL-6, IL-8, and TNF-α induced by each dissolving solution were defined as daily IL-6, IL-8, and TNF-α levels.

### 2.2. Statistical Analysis

To account for associations among repeated measurements within a subject, generalized estimating equation (GEE) logistic regression analyses were used to estimate the associations among the daily respiratory symptom of children and the daily levels of SPM, PM_2.5_, IL-6, IL-8, and TNF-α [31,32]. A respiratory symptom event was defined as the daily respiratory score ≥2. The GEE logistic regression models included individual characteristics (sex, height, weight, asthma, allergic rhinitis, allergic conjunctivitis, atopic dermatitis, and food allergies), meteorological variables such as daily temperature, humidity, and atmospheric pressure, and gaseous air pollutions (SO_2_, NO_2_, and ozone) [28,33,34,35]. Estimates are given as the odds ratio in respiratory symptom events per interquartile range (IQR) change of SPM and PM_2.5_ exposure, or IL-6, IL-8, and TNF-α levels, with 95% confidence intervals (CIs). The working correlation matrix was set to exchangeable, and the robust variance estimators were adopted for constructing CIs of the odds ratios. For treating missing data, the analysis used the multiple imputation method, which adequately addresses the uncertainty of imputed values based on multiply generated prediction values for missing data [36]. GEE analyses were performed using R version 3.2.2 (R Foundation for Statistical Computing, Vienna, Austria). The Pearson’s correlation coefficient analysis was conducted to estimate the associations among SPM, PM_2.5_, IL-6, IL-8, and TNF-α levels using SPSS software (Japanese version 20.0 for windows; IBM SPSS Japan Inc., Tokyo, Japan). All quoted *p*-values are two-sided, with a significance level of 0.05.

## 3. Results

### 3.1. Subject Characteristics

A total of 360 children were recruited, and their characteristics are shown in Table 1. Data was missing for height (*n* = 7) and body weight (*n* = 3).

### 3.2. SPM and PM_2.5_ Levels and the Associations between SPM, PM_2.5_, IL-6, IL-8, and TNF-α Levels

**Figure 1:**
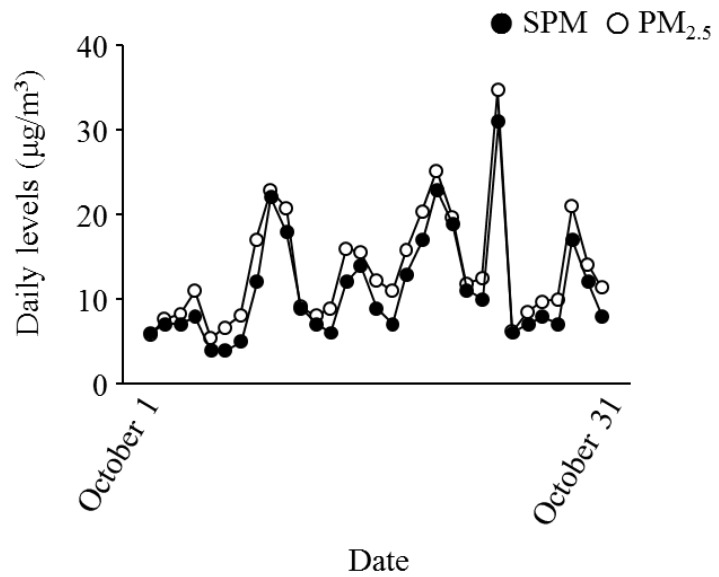
Daily average levels of SPM (closed circles) and PM_2.5_ (open circles). SPM = suspended particulate matter, PM_2.5_ = particulate matter smaller than 2.5 μm aerodynamic diameters.

**Figure 2:**
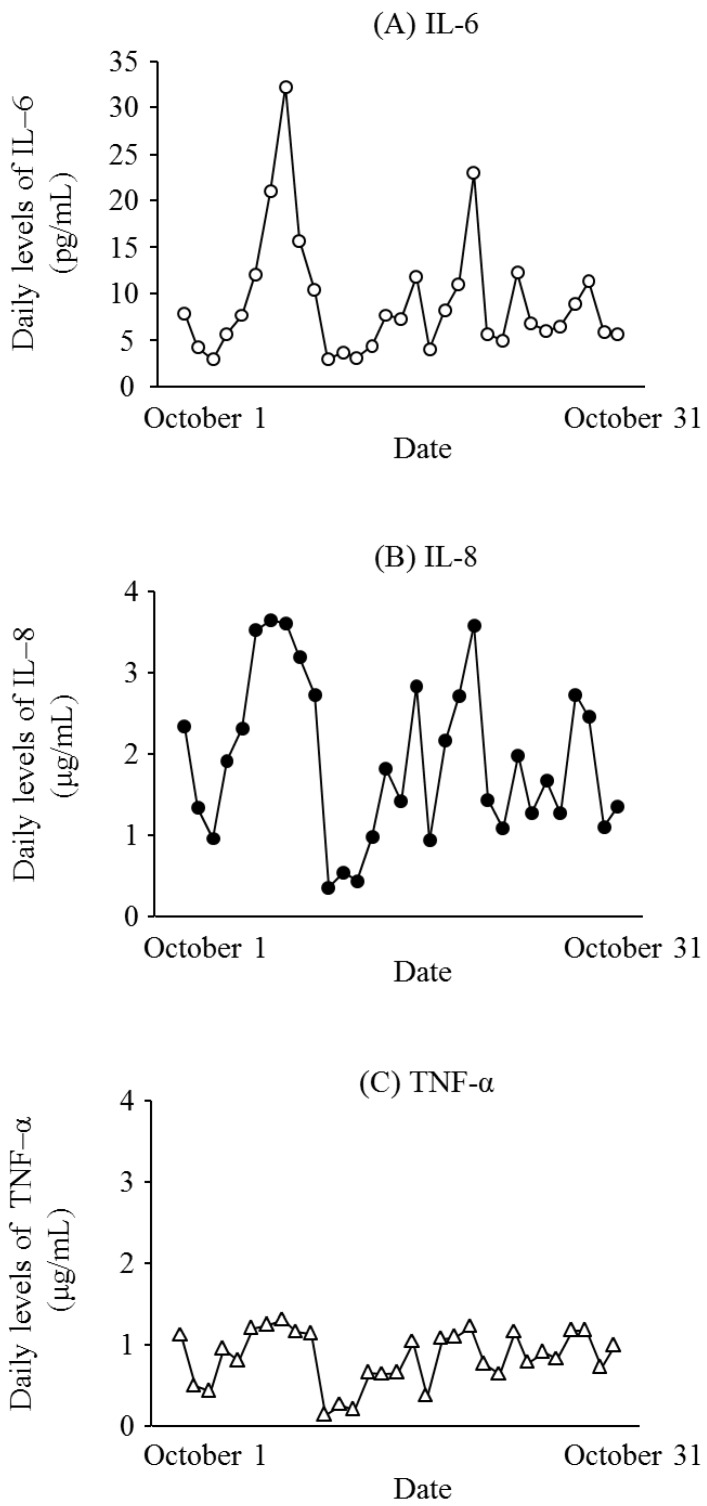
Daily levels of (**A**) interleukin (IL)-6 (open circles); (**B**) IL-8 (closed circles); and (**C**) tumor necrosis factor (TNF)-α(open triangle) for October 2015.

**Figure 3:**
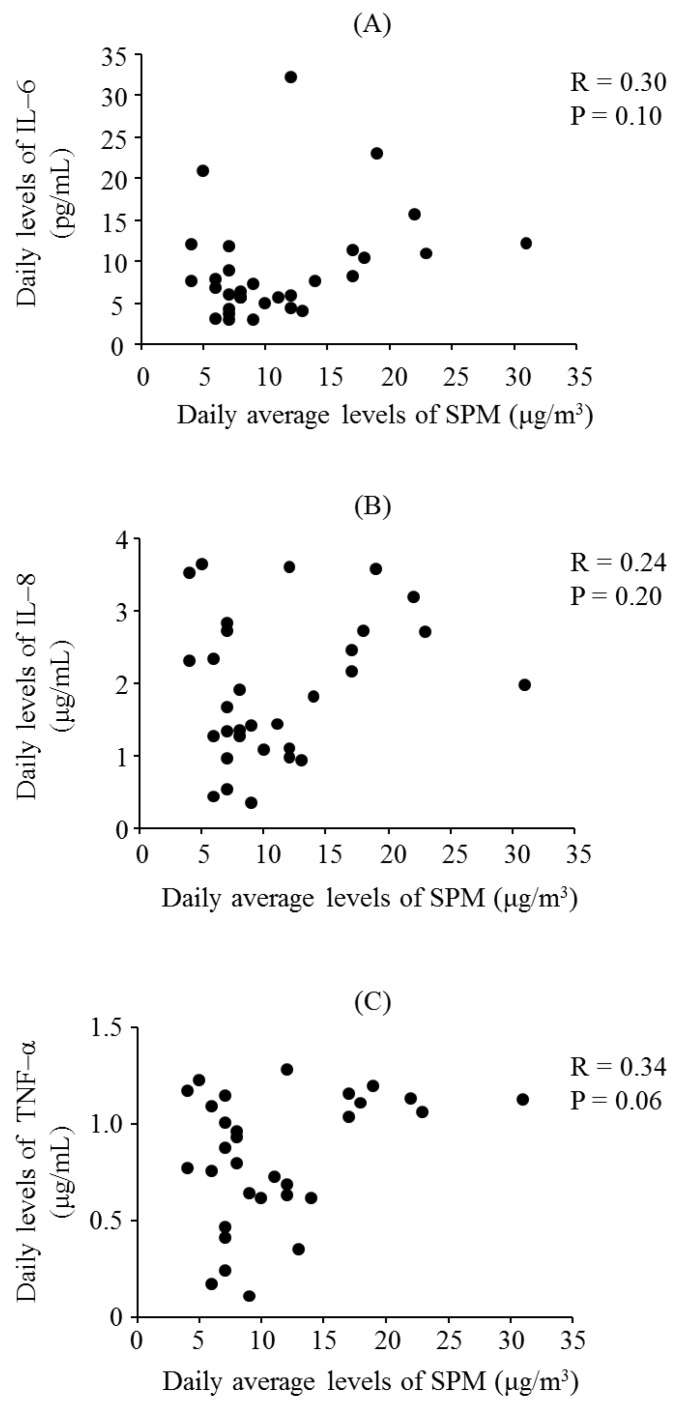
Associations between the daily levels of suspended particulate matter (SPM) and (**A**) interleukin (IL)-6; (**B**) IL-8; (**C**) tumor necrosis factor (TNF)-α.

**Figure 4:**
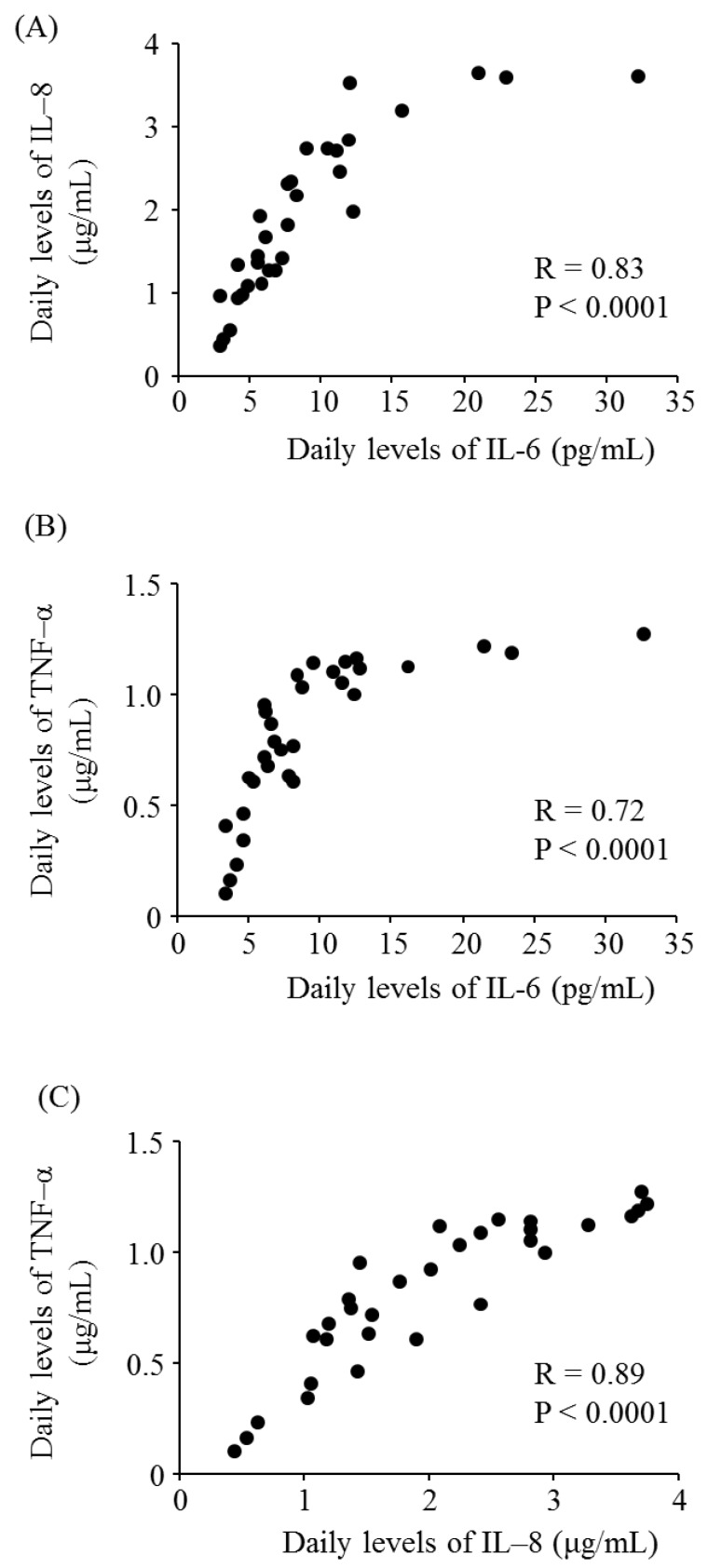
Associations among the daily levels of pro-inflammatory cytokines produced by THP1 human monocyte cells in response to stimulation with particulate matter. (**A**) Correlation of daily interleukin (IL)-6 and IL-8 levels; (**B**) Correlation of daily IL-6 and tumor necrosis factor (TNF)-α levels; and (**C**) Correlation of daily IL-8 and TNF-α levels.

Figure 1 presents the daily levels of SPM, PM_2.5_, IL-6, IL-8, and TNF-α for October 2015. The mean levels of SPM and PM_2.5_ during the study period were 10.8 ± 5.9 μg/m^3^ and 12.6 ± 6.4 μg/m^3^, respectively. The median levels of SPM and PM_2.5_ were 9.0 μg/m^3^ and 11.3 μg/m^3^, respectively. Figure 2 shows the daily levels of IL-6, IL-8, and TNF-α for October 2015. Figure 3 presents the associations between the daily levels of IL-6, IL-8, and TNF-α and the daily average levels of SPM from 1 October 2015 to 31 October 2015. Notably, the daily average SPM levels were not significantly associated with the daily levels of IL-6, IL-8, and TNF-α. There were significant correlations among the daily levels of IL-6, IL-8, and TNF-α (Figure 4).

### 3.3. Respiratory Symptoms

Table 2 shows the odds ratios in respiratory symptoms for an IQR increase in the levels of SPM, PM_2.5_, IL-6, IL-8, and TNF-α. Increases in the levels of SPM and PM_2.5_ were not related to an increased risk of respiratory symptom events, with increases of 6.0 μg/m^3^ SPM and 7.6 μg/m^3^ PM_2.5_ increasing odds ratios by 0.97 and 0.96, respectively. IL-6, IL-8, and TNF-α levels were significantly associated with increased respiratory symptom events. An increase of 6.16 pg/mL IL-6, 1.46 μg/mL IL-8, and 0.47 μg/mL TNF-α increased the odds ratios by 1.05, 1.12, and 1.11, respectively.

## 4. Discussion

To estimate the relationship between respiratory symptoms in schoolchildren and particulate air pollutants, the present study investigated the associations among daily SPM levels, daily IL-6, IL-8, and TNF-α levels, and respiratory symptoms for October 2015. While the daily levels of SPM were not associated with respiratory symptoms, the daily levels of IL-6, IL-8, and TNF-α showed a significant association with respiratory symptoms in schoolchildren.

Several reviews and meta-analyses have demonstrated the effects of particulate air pollutants on respiratory symptoms and pulmonary function in children, especially for children with asthma and some types of respiratory symptoms, although the results of these studies showed considerable heterogeneity [6,7,8]. In this study, the majority of participants did not have asthma and respiratory symptoms. Compared with other studies [6,7,8], this study had a large sample size. Therefore, the results of this study suggested that particulate air pollutants had little or no effect on respiratory symptoms in children, especially in children without respiratory disorders. In contrast, Weinmayr et al. indicated that the effects of PM_10_ on respiratory symptoms were increased during the summer and outside of Europe [6]. This may suggest that the effects of particulate air pollutant exposure on respiratory symptoms in schoolchildren differ according to the types and sources of PM. Our previous studies found variations in the effect of particulate air pollutants on pulmonary function in schoolchildren [25,26], and we demonstrated that the effects of particulate air pollutants on pulmonary function were more dependent on pro-inflammatory responses to specific particular air pollutants than on the mass concentration of pollutants [17]. In the present study, the injurious effects of particulate air pollutants on respiratory symptoms were associated with the production of pro-inflammatory cytokines. Daily average SPM levels were not significantly associated with the daily levels of IL-6, IL-8, and TNF-α. Only when particulate air pollutants have a high ability for inducing the pro-inflammatory response, depending on their composition, can they aggravate the respiratory symptoms of children.

Particulate air pollutants consist of various components, including elemental and organic carbon, elements, and ions, from several different sources, such as crustal material, traffic, biomass combustion, waste incineration, industrial processes, transported air pollution, road abrasion and resuspension, car brake debris, and bacteria and fungi dusts [37,38]. Therefore, it is important to determine the constituents and components of particulate air pollutants that are the most harmful to human health, including the respiratory system. Recently, Thurston et al. demonstrated that particulate air pollutants from fossil fuel combustion, especially coal burning—but also from diesel traffic—were most strongly associated with increases in ischemic heart disease mortality compared with components from other sources [39]. However, most of the components in particulate air pollutants showed strong adverse effects on human health [40]. The constituents and components of particulate air pollutants that are most injurious to human health have yet to be defined. To determine which constituents and components of particulate air pollutants are most harmful for human health, it may be useful to investigate which components have a strong potential for production of pro-inflammatory cytokines. In the present study, we were unable to analyze the components of the collected airborne particles. Further studies are required to determine the components with a strong potential for inducing the production of pro-inflammatory cytokines.

The daily amount of airborne PM was too small compared with the filter weight, and the quantity of daily collected airborne PM lacked precision. Therefore, it was concluded that calculation of the IL-6, IL-8, and TNF-α levels per unit quantity of collected airborne PM was not suitable for this study. Instead, the collected airborne PM was dissolved in 4 mL distilled deionized water and the concentrations of IL-6, IL-8, and TNF-α produced by THP1 cells stimulated with the solution of PM were measured and expressed as daily levels. This method seemed appropriate to investigate the relationship between respiratory symptoms and particulate air pollutant-related IL-6, IL-8, and TNF-α production, as the amount of inhaled particulate air pollutants is related to SPM levels.

Asthma is characterized by respiratory symptoms, including wheezing, dyspnea, a cough, and sputum, which are dependent on chronic airway inflammation, airway hyperresponsiveness, and airway remodeling [41]. Compared with children without asthma, children with asthma easily show respiratory symptoms in response to inhalation of various environmental factors, such as air pollution, allergens, and dust [41]. Nasopharyngeal dysfunctions, including allergic rhinitis, play an important role in bronchoconstriction [42]. Therefore, the present study adjusted for allergic diseases when investigating the association between air pollutants and respiratory symptoms.

According to the manufacturer’s protocol, higher concentrations of proteins such as IL-6, IL-8, and TNF-α are associated with higher measurement errors. This is because the ELISA kit calculates the concentrations of proteins using a standard curve depending on the absorbance; when the value approaches the upper limit of the measuring range, the value tends to be underestimated. Compared with IL-6, the concentration ranges of IL-8 and TNF-α were wide in the present study. Therefore, when IL-6 levels exceeded 15 pg/mL, IL-6 was not associated with the levels of IL-8 and TNF-α because the levels of IL-8 and TNF-α had reached the upper limits of their capable ranges. Thus, it may have been unsuitable to estimate the association of IL-6 with IL-8 and TNF-α when IL-6 levels exceeded 15 pg/mL in the present study.

The IQR of SPM was less than that of PM_2.5_ during this study period, which was an inexplicable phenomenon. The same phenomenon was also observed in another Japanese study [43]. We consulted with the Japanese Ministry of the Environment regarding this discrepancy, who suggested that the discrepancy was caused by different measurement principles for different measuring devices.

This study had several limitations. First, the study duration, which was one month, may not have been long enough to estimate the relationship between particulate air pollutants and respiratory symptoms, and to analyze the associations between SPM and PM_2.5_ and the production of IL-6, IL-8, and TNF-α. However, a few studies investigated the relationship between particulate air pollutants and respiratory function in a study period that was less than one month [8,44]. The number of study participants was large, as compared with other studies [8]. Therefore, we considered our results to be reliable. Second, we filtered the extraction liquids of collected airborne PM through 10-μm filters to remove PM > 10 μm in diameter. Water soluble components attached to PM > 10 μm were also included in the extraction liquid for measurement. The IL-6, IL-8, and TNF-α levels in this study may be reflected with PM > 10 μm. However, PM > 10 μm is also inhaled into the lower respiratory tract via mouth breathing [45]. Therefore, this limitation did not likely affect the results. Third, missing respiratory symptom scores due to absences from school were excluded from the data analysis. This intermittent missing data would be statistically independent and did not cause any serious biases in the results. However, previous studies reported that air pollution was associated with increased hospital admissions and emergency visits [7,8]. Therefore, the missing data corresponding to absences in this study may have been due to hospital admissions and/or emergency visits caused by high levels of air pollution. As such, the present study may have underestimated the adverse effects of particulate air pollutants on respiratory symptoms in children. Finally, we were unable to measure the individual amount of exposure to particulate air pollutants.

## 5. Conclusions 

Short-term Exposure to SPM and PM_2.5_ was not associated with respiratory symptoms in schoolchildren. However, this study found a significant association between respiratory symptoms in schoolchildren and the daily levels of IL-6, IL-8, and TNF-α induced by collected airborne PM. Only when particulate air pollutants have a high ability for inducing the pro-inflammatory response, which is dependent on the types and sources of PM, are they able to aggravate the respiratory symptoms of children. 

## Figures and Tables

**Table 1:** Characteristics of the 360 children included in this study.

Characteristic		Value
Boy/Girl		172/188
Age (Years)	11	139 (38.6)
	12	220 (61.1)
	13	1 (0.3)
Height (cm)		148.6 ± 7.4
Weight (kg)		39.0 ± 7.7
Allergic Disease	Sthma	39 (10.8)
	Allergic Rhinitis	72 (20.0)
	Allergic Conjunctivitis	10 (2.8)
	Atopic Dermatitis	26 (7.2)
	Food Allergies	15 (4.2)

Data are shown as the mean ± standard deviation or *n* (%). Data were missing for height (*n* = 7) and body weight (*n* = 3).

**Table 2:** Multivariate analysis using generalized estimating equation (GEE) logistic regression models to assess the association between respiratory symptoms and interquartile range (IQR) changes in the levels of suspended particulate matter (SPM), particulate matter <2.5 μm in diameter (PM_2.5_), interleukin (IL)-6, IL-8, and tumor necrosis factor (TNF)-α.

Exposure Metric	IQR	Odds Ratio	95% CI	*p*-Value
SPM	6.0 μg/m^3^	0.97	0.91–1.02	NS
PM_2.5_	7.6 μg/m^3^	0.96	0.91–1.02	NS
IL-6	6.16 pg/mL	1.05	1.01–1.09	0.02
IL-8	1.46 μg/mL	1.12	1.04–1.20	0.002
TNF-α	0.47 μg/mL	1.11	1.03–1.19	0.004

CI = confidence interval; NS = not significant.
